# Dietary Iron, Anemia Markers, Cognition, and Quality of Life in Older Community-Dwelling Subjects at High Cardiovascular Risk

**DOI:** 10.3390/nu15204440

**Published:** 2023-10-19

**Authors:** Carolina Donat-Vargas, Víctor Mico, Rodrigo San-Cristobal, Miguel Ángel Martínez-González, Jordi Salas-Salvadó, Dolores Corella, Montserrat Fitó, Ángel Maria Alonso-Gómez, Julia Wärnberg, Jesús Vioque, Dora Romaguera, José López-Miranda, Ramon Estruch, Miguel Damas-Fuentes, José Lapetra, Luís Serra-Majem, Aurora Bueno-Cavanillas, Josep Antoni Tur, Sergio Cinza-Sanjurjo, Xavier Pintó, Miguel Delgado-Rodríguez, Pilar Matía-Martín, Josep Vidal, Claudia Causso, Emilio Ros, Estefanía Toledo, Josep Maria Manzanares, Carolina Ortega-Azorín, Olga Castañer, Patricia Judith Peña-Orihuela, Juan Manuel Zazo, Carlos Muñoz Bravo, Diego Martinez-Urbistondo, Alice Chaplin, Rosa Casas, Naomi Cano Ibáñez, Lucas Tojal-Sierra, Ana María Gómez-Perez, Elena Pascual Roquet-Jalmar, Cristina Mestre, Rocío Barragán, Helmut Schröder, Antonio Garcia-Rios, Inmaculada Candela García, Miguel Ruiz-Canela, Nancy Babio, Mireia Malcampo, Lidia Daimiel, Alfredo Martínez

**Affiliations:** 1 ISGlobal, Campus Mar, 08036 Barcelona, Spain; 2 CIBER de Epidemiología y Salud Pública (CIBERESP), Instituto de Salud Carlos III, 28029 Madrid, Spain; vioque@umh.es (J.V.); abueno@ugr.es (A.B.-C.); ncaiba@ugr.es (N.C.I.); hschoeder@imim.es (H.S.); 3 Cardiometabolic Nutrition Group, IMDEA Food, CEI UAM + CSIC, 28049 Madrid, Spain; victor.mico@imdea.org (V.M.); rodrigo.san-cristobal-blanco.1@ulaval.ca (R.S.-C.); mdelgado@ujaen.es (M.D.-R.); 4 Biomedical Research Centre for Obesity Physiopathology and Nutrition Network (CIBEROBN), Instituto de Salud Carlos III (ISCIII), 28029 Madrid, Spain; mamartinez@unav.es (M.Á.M.-G.); dolores.corella@uv.es (D.C.); mfito@imim.es (M.F.); angelmago13@gmail.com (Á.M.A.-G.); jwarnberg@uma.es (J.W.); mariaadoracion.romaguera@ssib.es (D.R.); jlopezmir@gmail.com (J.L.-M.); restruch@clinic.ub.es (R.E.); migueldamasf@hotmail.com (M.D.-F.); joselapetra543@gmail.com (J.L.); lserra@dcc.ulpgc.es (L.S.-M.); pep.tur@uib.es (J.A.T.); xpinto@bellvitgehospital.cat (X.P.); eros@clinic.cat (E.R.); etoledo@unav.es (E.T.); carolina.ortega@uv.es (C.O.-A.); patricia.pena@imibic.org (P.J.P.-O.); alicemarylillian.chaplin@ssib.es (A.C.); rcasas1@clinic.cat (R.C.); lutojal@hotmail.com (L.T.-S.); anamgp86@gmail.com (A.M.G.-P.); cristinamestressola@gmail.com (C.M.); rocio.barragan@uv.es (R.B.); h72garia@uco.es (A.G.-R.); mcanela@unav.es (M.R.-C.); nancy.babio@urv.cat (N.B.); 5 Department of Preventive Medicine and Public Health, University of Navarra, IDISNA, 31008 Pamplona, Spain; 6 Department of Nutrition, Harvard T. H. Chan School of Public Health, Boston, MA 02115, USA; 7 Unitat de Nutrició Humana, Departament de Bioquímica i Biotecnologia, Universitat Rovira i Virgili, 43201 Reus, Spain; jordi.salas@urv.cat (J.S.-S.); josema.manzanares@salutsantjoan.cat (J.M.M.); 8 Food, Nutrition, Development and Mental Health Research Group, Institut d’Investigació Pere Virgili (IISPV), 43204 Reus, Spain; 9 Department of Preventive Medicine, University of Valencia, 46010 Valencia, Spain; 10 Unit of Cardiovascular Risk and Nutrition, Institut Hospital del Mar de Investigaciones Médicas Municipal d’Investigació Médica (IMIM), 08003 Barcelona, Spain; ocastaner@imim.es (O.C.); mmalcampo@imim.es (M.M.); 11 Bioaraba Health Research Institute, Osakidetza Basque Health Service, Araba University Hospital, University of the Basque Country UPV/EHU, 01009 Vitoria-Gasteiz, Spain; 12 EpiPHAAN Research Group, Department of Nursing, School of Health Sciences, University of Málaga-IBIMA (Instituto de Investigación Biomédica de Málaga), 29071 Málaga, Spain; 13 Instituto de Investigación Sanitaria y Biomédica de Alicante, Universidad Miguel Hernández (ISABIAL-UMH), 03010 Alicante, Spain; 14 Health Research Institute of the Balearic Islands (IdISBa), 07120 Palma de Mallorca, Spain; 15 Lipids and Atherosclerosis Unit, Department of Internal Medicine, Maimonides Biomedical Research Institute of Cordoba (IMIBIC), Reina Sofia University Hospital, University of Cordoba, 14004 Córdoba, Spain; 16 Department of Internal Medicine, Institut d’Investigació Biomèdica August Pi I Sunyer (IDIBAPS), Hospital Clinic, University of Barcelona, 08036 Barcelona, Spain; 17 Department of Endocrinology, Instituto de Investigación Biomédica de Málaga (IBIMA), Virgen de la Victoria Hospital, University of Málaga, 29016 Málaga, Spain; 18 Department of Family Medicine, Research Unit, Distrito Sanitario Atención Primaria Sevilla, 41013 Sevilla, Spain; 19 Research Institute of Biomedical and Health Sciences (IUIBS), University of Las Palmas de Gran Canaria, Preventive Medicine Service, Centro Hospitalario Universitario Insular Materno Infantil (CHUIMI), Canarian Health Service, 35016 Las Palmas, Spain; 20 Department of Preventive Medicine and Public Health, University of Granada, 18016 Granada, Spain; 21 Research Group on Community Nutrition & Oxidative Stress, University of Balearic Islands, 07122 Palma de Mallorca, Spain; 22 CS Milladoiro, Área Sanitaria de Santiago de Compostela, 15706 Santiago de Compostela, Spain; scinzas@semergen.es; 23 Instituto de Investigación de Santiago de Compostela (IDIS), 15706 Santiago de Compostela, Spain; 24 Departamento de Medicina, Universidad de Santiago de Compostela, 15701 Santiago de Compostela, Spain; 25 Lipids and Vascular Risk Unit, Internal Medicine, Hospital Universitario de Bellvitge, Hospitalet de Llobregat, 08907 Barcelona, Spain; 26 Division of Preventive Medicine, Faculty of Medicine, University of Jaén, 23071 Jaén, Spain; 27 Department of Endocrinology and Nutrition, Instituto de Investigación Sanitaria Hospital Clínico San Carlos (IdISSC), 28040 Madrid, Spain; pilar.matia@gmail.com; 28 Department of Endocrinology, IDIBAPS, Hospital Clinic, University of Barcelona, 08036 Barcelona, Spain; jovidal@clinic.cat; 29 Biomedical Research Centre for Diabetes and Metabolic Diseases Network (CIBERDEM), Instituto de Salud Carlos III (ISCIII), 28029 Madrid, Spain; 30 Servicio de Endocrinologia Hospital General de Villalba, 28400 Madrid, Spain; claudiamcausso@gmail.com; 31 Lipid Clinic, Department of Endocrinology and Nutrition, Institut d’Investigacions Biomèdiques August Pi Sunyer (IDIBAPS), Hospital Clínic, 08036 Barcelona, Spain; 32 Department of Preventive Medicine and Public Health, School of Medicine, Instituto de Investigación Biomédica de Málaga, University of Málaga, 29590 Málaga, Spain; juanmazazo@gmail.com; 33 Department of Public Health and Psychiatry, University of Malaga-IBIMA (Instituto de Investigación Biomédica de Málaga), 29071 Málaga, Spain; 34 Internal Medicine Department, Clínica Universidad de Navarra, 28022 Madrid, Spain; dmurbistondo@gmail.com; 35 Instituto de Investigación Biosanitaria ibs.Granada, 18012 Granada, Spain; 36 Osasunbidea, Servicio Navarro de Salud, 31003 Pamplona, Spain; elena.pascual.roquetjalmar@navarra.es; 37 Primary Health Care Center Santa Pola, 03130 Alicante, Spain; candelinma@gmail.com; 38 Nutritional Control of the Epigenome Group, Precision Nutrition and Obesity Program, IMDEA Food, CEI UAM + CSIC, 28049 Madrid, Spain; lidia.daimiel@imdea.org; 39 Department of Nutrition, Food Sciences and Physiology, University of Navarra, 31008 Pamplona, Spain

**Keywords:** cognition, iron, anemia, diabetes, quality of life, epidemiology, older adults

## Abstract

Anemia causes hypo-oxygenation in the brain, which could lead to cognitive disorders. We examined dietary iron intake as well as anemia markers (i.e., hemoglobin, hematocrit, mean corpuscular volume) and diabetes coexistence in relation to neuropsychological function and quality of life. In this study, 6117 community-dwelling adults aged 55–75 years (men) and 60–75 years (women) with overweight/obesity and metabolic syndrome were involved. We performed the Mini-Mental State Examination (MMSE), the Trail Making Test parts A and B (TMT-A/B), Semantic Verbal Fluency of animals (VFT-a), Phonological Verbal Fluency of letter P (VFT-p), Digit Span Test (DST), the Clock Drawing Test (CDT), and the Short Form-36 Health Survey (SF36-HRQL test). Dietary iron intake did not influence neuropsychological function or quality of life. However, anemia and lower levels of anemia markers were associated with worse scores in all neurophysiological and SF36-HRQL tests overall, but were especially clear in the MMSE, TMT-B (cognitive flexibility), and the physical component of the SF36-HRQL test. The relationships between anemia and diminished performance in the TMT-A/B and VFT tasks were notably pronounced and statistically significant solely among participants with diabetes. In brief, anemia and reduced levels of anemia markers were linked to inferior cognitive function, worse scores in different domains of executive function, as well as a poorer physical, but not mental, component of quality of life. It was also suggested that the coexistence of diabetes in anemic patients may exacerbate this negative impact on cognition. Nevertheless, dietary iron intake showed no correlation with any of the outcomes. To make conclusive recommendations for clinical practice, our findings need to be thoroughly tested through methodologically rigorous studies that minimize the risk of reverse causality.

## 1. Introduction

As life expectancy increases, the world’s elderly population is also rising. Therefore, preserving both cognitive function and quality of life is becoming a main challenge for health care professionals [1]. At the same time, lower hemoglobin levels are often seen with advancing age, especially after 65 years [2]. According to the World Health Organization (WHO), anemia is defined as hemoglobin levels below 13 g/dL in men and 12 g/dL in women [3]. Based on these limits, anemia in older adults is a frequent, overlooked, and potentially complicating morbid condition [4] that represents a major cause of years lived with disability [5]. 

Iron proteins have key roles in normal brain function and the processes of brain development. Thus, anemia significantly hampers brain development, leading to reduced cognitive abilities and impaired learning in children and adolescents [6]. In adults, anemia may cause hypo-oxygenation in the brain, which can trigger cognition disturbances and, ultimately, neurological manifestations including dementia [7]. Evidence from observational studies suggest that anemia is associated with cognitive impairment and dementia [8]. Even hemoglobin levels slightly below the WHO anemia threshold have shown a substantial negative impact on the quality of life of older adults [9]. Likewise, individuals with type 2 diabetes face an estimated two- to three-fold higher risk of anemia than those individuals without diabetes [10]. In turn, diabetes itself increases the risk of cognitive dysfunction [11].

On the other hand, iron accumulation in the brain occurs during the aging process in humans, as well as in neurodegenerative diseases, due to dysregulation in iron homeostatic mechanisms [12]. While some studies suggest that a high dietary iron intake might negatively affect cognition and contribute to neurodegeneration, our understanding of the effects of elevated iron intake remains limited [13,14]. Generally, studies in humans investigating the relationship between dietary and metabolic iron and cognition and quality of life are scarce, and the results are inconclusive. The interaction between red blood cells and diabetes status in relation to cognition and quality of life also deserves further epidemiological evidence. 

Therefore, to explore the associations between dietary iron intake, the status of iron metabolism within the body, anemia, prevalent diabetes, neuropsychological functions, and quality of life, we undertook a cross-sectional study among older residents of Spanish communities who exhibited an elevated risk of cardiovascular disease. 

## 2. Materials and Methods

### 2.1. Study Design and Participants

Eligible participants for the present study were community-dwelling adults aged between 55 and 75 years in the case of men and between 60 and 75 years in women. They had overweight or obesity (body mass index (BMI) between 27 and 40 kg/m^2^) with metabolic syndrome (according to the International Diabetes Federation and the American Heart Association) but without cardiovascular disease. The study protocol recruitment flowchart and the specific exclusion criteria were described extensively elsewhere [15]. Briefly, participants were recruited from October 2013 to December 2016 across 23 centers from different universities, hospitals, and research institutes in Spain. A total of 6609 candidates met eligibility criteria. Participants provided data on cognitive performance, quality of life, anthropometrical and biochemical measures, physical conditions, and sociodemographic parameters.

All participants provided written informed consent, and the study protocol and procedures were approved according to the ethical standards of the Declaration of Helsinki by the Research Ethics Committees from all the participating institutions. This study was registered with the International Standard Randomized Controlled Trial (ISRCT; http://www.isrctn.com/ISRCTN89898870, accessed on 2 August 2023) with number 89898870.

### 2.2. Exposure Assessment

After fasting overnight, blood samples were collected and frozen (−80 °C) at the initial screening visits. Standard red cell indices that are typically examined as part of a routine checkup for diagnosing and evaluating anemia were assessed. These include hemoglobin (g/dL), hematocrit (%), and mean corpuscular volume (MCV, fL). These markers were chosen for their ease of measurement via a straightforward blood test and cost-effectiveness. These blood cells are direct indicators of the blood’s oxygen-carrying capacity and are used to diagnose anemia. Anemia was defined according to the World Health Organization (WHO) criteria of hemoglobin concentrations below 13 g/dL for men and below 12 g/dL for women.

Total and heme dietary iron intake were also estimated by combining data from the annually administered food frequency questionnaire (FFQ) validated for Spanish populations [16] and conventional food composition tables. We used the residual method to adjust dietary iron intake for total energy intake by regressing iron intake on total energy intake derived from the FFQ.

### 2.3. Measurement of Cognitive and Executive Functions

A battery of eight neurophysiological tests were administered at baseline by trained staff. Given its rapid and straightforward administration by health care professionals, cognitive function was evaluated using a Spanish validated version of the Mini-Mental State Examination (MMSE). This 30-question assessment is a widely used screening tool in clinical and research settings to evaluate cognitive functioning, specifically focusing on cognitive impairment and dementia. The MMSE evaluates various cognitive domains, including orientation, registration, attention and calculation, recall, language, visual–spatial abilities, and executive functioning. Higher scores indicate better cognitive function [17,18]. Additionally, we used several other common neuropsychological tests to evaluate a person’s executive functions, i.e., ability to plan, organize, initiate, inhibit, and monitor their behavior. The neuropsychological tests were the following: the Trail Making Test parts A (TMT-A, assesses attention and processing speed capacities) and B (TMT-B, assesses cognitive flexibility) [19]; Semantic Verbal Fluency of animals (VFT-a) and Phonological Verbal Fluency of letter P (VFT-p) (both assess verbal ability and executive function) [20]; Digit Span Test forward (DST-f, assesses immediate memory) and backward (DST-b, assesses working memory function) [21]; and the Clock Drawing Test (CDT, assesses visuospatial, visuoconstruction, and memory capacities, as well as verbal and numerical knowledge) [22]. Complete explanations of these tests can be found in the Supplementary Materials File S1.

### 2.4. Health-Related Quality of Life

To comprehensively evaluate health-related quality of life (HRQL), a survey was performed with an adapted version of the previously published Short Form-36 Health Survey (SF-36), which was completed during the baseline visit. This SF36-HRQL test has been validated for the Spanish population [23,24] and consists of 8 health concept domains: physical function (PF), role limitations due to physical health problems (RP), bodily pain (BP), general health (GH), vitality (VT), social functioning (SF), role limitations due to emotional problems (RE), and mental health (MH). From these individual subscales, two aggregated scores were generated: physical health component (PCS) and mental health component (MCS). The first five subscales (PF, RP, BP, GH, VT) produce the PCS and the last five subscales (GH, VT, SF, RE, MH) produce the MCS; the GH and VT subscales overlap between the two aggregated components. The scorings for the eight domains and the two aggregated components are based on a 0 to 100 scale, with a higher value corresponding to a better HRQL. This SF36-HRQL test has been extensively used in Spain as an accurate way to measure generic self-perceived HRQL [25].

### 2.5. Covariates

Covariates were evaluated by trained staff in a face-to-face interview using self-reported general questionnaires on socio-demographics (sex, age, level of education, and civil status), lifestyle (alcohol intake, smoking habits, physical activity, and Mediterranean diet adherence), and history of disease. Adherence to the Mediterranean diet was assessed using a previously validated 17-point questionnaire score [26]. Leisure-time physical activity was estimated using a validated Regicor Short Physical Activity Questionnaire for the Adult Population [27]. Depressive status risk was evaluated using the Beck Depression Inventory-II [28].

Blood lipid levels were measured via routine laboratory tests using standardized enzymatic methods. Blood pressure was measured using a validated semiautomatic oscillometer (Omron HEM-705CP, ’s-Hertogenbosch, The Netherlands) after 1 min of rest in-between measurements. Hypercholesterolemia was defined as fasting plasma total cholesterol level ≥ 200 mg/dL or taking lipid-lowering medications; high blood pressure was ascertained when systolic ≥ 131 and diastolic ≥ 81 mmHg or taking antihypertensive medication; and diabetes was defined as glucose ≥ 126 mg/dL or taking diabetes medication. Height, waist circumference, and weight were measured at baseline by trained staff. BMI was calculated as weight (kg) over height squared (m^2^). All anthropometric variables were determined in duplicate, except for blood pressure (in triplicate).

### 2.6. Statistical Analysis

The June 2020 database of this study was used in all analyses. Descriptive variables are reported as means and standard deviations (SD) for continuous variables or numbers and percentages (%) for qualitative variables. Differences between anemia status and baseline characteristics were examined using Pearson’s chi-square test for categorical variables and one-way ANOVA test for continuous variables.

Adjusted linear regression models, estimating beta coefficients (i.e., mean differences), and 95% confidence intervals (95% CI) were used to cross-sectionally assess the association of (i) anemia status (as dichotomic variable), (ii) levels of different anemia markers (i.e., hemoglobin, hematocrit, and MCV), as well as (iii) total and heme dietary iron intake (categorized in tertiles) with the different neurophysiological tests evaluating the cognitive function (Mini-Mental test), the executive function (TMT, VFT-a, VFT-p, DST), and HRQL (SF36-HRQL test), differentiating by physical and mental dimensions.

To elucidate the role that diabetes may play in the relationship between iron and neurodegeneration, associations were further stratified by prevalent diabetes and statistical interactions were tested for each subgroup analysis using the likelihood ratio test of models with and without the interaction term. 

We adjusted the regression models for several potential confounders defined a priori and selected according to previous causal knowledge. Two gradual models were built with progressive levels of adjustment for confounders based on information collected at enrollment. Model 1 was adjusted for baseline: sex, age (years), vitamin B12 (mcg/d) and folate (mcg/d) intake, and recruitment center. Model 2 was additionally adjusted for baseline BMI (kg/m^2^), education level (primary, secondary, or college), tobacco use (current, never, or former smoker), physical activity (METs min/week), adherence to a Mediterranean diet (score of 17 points), hours of sleep (hours/d), alcohol consumption (g/d), systolic and diastolic blood pressure levels (mmHg), cholesterol levels (mg/dL), and prevalent cancer, depression, and diabetes. Missing values in some variables were always less than 0.7% and were imputed using stochastic regression imputation. This approach involves estimating and filling in missing values by modeling the relationships between variables through regression analysis, but with an added element of randomness or uncertainty (stochasticity). This extra step of augmenting each predicted score with a residual term provides a more accurate representation of the missing data. 

The data were analyzed using the Stata 16 software program (StataCorp LP, College Station, TX, USA) and statistical significance was set at a two-tailed *p* value < 0.05.

## 3. Results

Out of the 6609 recruited participants, 6602 were free of dementia and, among these, 6117 had complete data on dietary and anemia markers. The subjects included had a mean age of 66 (±5) years, and 252 (4.1%) had anemia. On average, those with anemia were around 1 year older, more frequently women, had higher fasting glucose levels, consumed less alcohol, had a lower educational level, were less frequently current smokers, had higher BMI levels, and more frequently presented type 2 diabetes compared with subjects with no anemia (Table 1).

On average, anemic subjects had worse crude scores in all neurophysiologic and HRQL tests than non-anemic subjects. Mean differences were calculated adjusting for different sociodemographic and clinical variables, showing that scores for all neuropsychological and HRQL tests were maintained across anemic and non-anemic subjects, whereas the MMSE test, the TMT-B (cognitive flexibility), and the SF-36 aggregated physical component scored significantly worse (Table 2). Higher hemoglobin and hematocrit continuous levels were also significantly dose-response associated with these same tests (Supplementary Table S1). The MCV levels were only significantly associated with the SF-36 aggregated physical component score (Supplementary Table S1). In addition, both anemia and anemia markers were significantly associated with each individual physical component of the SF36-HRQL test, i.e., physical function, role limitations due to physical health problems, bodily pain, vitality, and general health (Supplementary Table S2).

When we analyzed the association between anemia status, cognitive and executive functions, and HRQL, differentiating between prevalent diabetes (Table 3), the associations of anemia with poorer cognitive function (MMSE test) and physical HRQL were similar in both subjects with diabetes and without. Nevertheless, associations between anemia and poor scores in the TMT and VFT (executive function and verbal ability) were stronger and only significant in participants with diabetes, and interactions between anemia and diabetes were identified. Overall, both total and heme iron intakes were not associated with any neuropsychological test or with HRQL (Table 4). Only the TMT (both A and B) scored significantly worse in those with a higher iron intake (Table 4).

## 4. Discussion

The aim of this study was to explore the link between levels of anemia markers, anemia status, and diabetes coexistence in relation to cognitive and executive functions and HRQL in older adults at risk of cardiovascular disease. The main findings from this research can be summarized as: (1) there was a consistent association of anemia and levels of hemoglobin and hematocrit with the MMSE test (general cognitive function), the TMT: B (cognitive flexibility), and the physical components of the SF-36 test (physical function, role limitations due to physical health problems, bodily pain, vitality, and general health); (2) anemia and anemia markers, however, were not associated with tests evaluating attention and memory, or with the mental components of the SF-36; (3) dietary iron intake was not correlated with anemia, and a higher iron intake was not associated with any neurophysiological or HRQL tests; (4) 7% of those diagnosed with diabetes had anemia compared with 3% of the non-anemic; (5) when stratifying by prevalent diabetes, significant associations of anemia status with both poorer cognitive function (MMSE) and diminished SF-36 physical component score remained in both subjects with diabetes and without; (6) however, associations between anemia and poor scores in the TMT and VFT (verbal ability/semantic memory) were stronger and only significant in diabetic participants with manifested interactions between anemia and diabetes. The association with the SF-36 physical component score was also slightly stronger in subjects with diabetes compared with those without.

Iron deficiency and heart failure often coexist, and the combination of the two conditions manifests with worse outcomes [29]. Causes of anemia include iron, folate, and vitamin B12 deficiencies, renal insufficiency, chronic inflammation (formerly termed anemia of chronic disease), and unexplained anemia (also named idiopathic anemia) [30]. It is estimated that in older persons with anemia, nutrient deficiency may be present in one-third, chronic disease or renal insufficiency or both may account for another third, and idiopathic anemia is present in the remaining third [31,32]. A significant group of older anemic patients remain untreated due to the lack of a known cause of the decrease in hemoglobin; hence, anemia can negatively affect health for many years [33]. In the participants of this study, anemia does not appear to be caused by lower dietary iron, folate, or vitamin B12 intake, and a lower iron intake was not associated with cognition impairment or decreased HRQL. Proposed mechanisms of anemia in older adults which are not blood loss/nutrition-related include dysregulation of inflammatory responses, blunting of the hypoxia/erythropoietin sensing mechanism, sarcopenia, quantitative/qualitative alterations in stem cell physiology, a decrease in sex steroids, frequent co-morbid medical conditions, and polypharmacy [30]. 

Previous studies conducted in community-based older people have already suggested a link between hemoglobin levels and anemia status with both cognitive decline and dementia [34]. Diminished mean corpuscular hemoglobin and red cell distribution width have also been associated with poorer cognitive performance [35]. Likewise, low hemoglobin levels were associated with decreased cortical thickness and brain volume [36]. A recent random-effects meta-analysis of 20 observational studies indicated that anemic patients have a nearly 1.39-fold risk of developing overall cognitive impairment than non-anemic subjects [34]. However, there is less consistent on the potential distinct impact of red cell index levels and anemia status in the different cognitive domains. A cross-sectional study conducted in community-dwelling older persons from the Rush Memory and Aging Project found that hemoglobin was related to semantic memory and perceptual speed but not to episodic memory, working memory, or visuospatial ability [37]. In older participants from the Epidemiology of Dementia in Singapore study, decreased hemoglobin levels were associated with worse performance in the attention and language domains [36]. Anemia has also been associated with slower processing speed, decreased working memory, and decreased intracranial volume on brain magnetic resonance imaging in a cross-sectional study involving mainly older African Americans [38]. Specifically, there is a previous study that found anemia to be associated with low performance on the TMT A and B [39]. Our analysis agrees with this precedent literature. 

The MMSE is a valid, routinely utilized instrument for the screening of global cognitive function focused on cortical functions such as memory [40], while the TMT gives information on the executive function domain that is relevant to mobility and autonomy in activities of daily living, involving abilities such as visual–conceptual and visual–motor tracking or sustained attention [41]. Current evidence suggests that executive function impairment might precede and causally mediate the onset and progression of functional decline [42]. In this sense, older community-dwelling adults with poor performance in the TMT showed they were at a higher risk of developing mobility impairments and experiencing accelerated decline of lower extremity performance and had higher risk of dying [42]. Therefore, executive function may be the critical “cognitive” component of mobility, which is particularly important when facing real-life mobility tasks that are complex and challenging [43]. 

Our findings support this connection between executive function and physical function, since we found that anemia markers mainly associated with both the TMT, which evaluates executive functions (including attention, processing speed capacities, and cognitive flexibility), and the physical, but not mental, dimension of the SF36-HRQL test.

Iron plays a crucial role in cellular processes such as energy metabolism, nucleotide synthesis, and numerous enzymatic reactions [44]. A decline in muscle mass is associated with a diminished quality of life and an increased risk of morbidity and premature mortality [44]. While the influence of iron deficiency on muscle mass and function is not yet fully understood, several studies indicate that iron deficiency may play a role in muscle mass reduction. In individuals within the general population, iron deficiency has been linked to reduced muscle mass, independently of hemoglobin levels [44]. Iron deficiency has also been shown to impair myoblast proliferation and aerobic glycolytic capacity while inducing markers of myocyte atrophy and apoptosis [44].

The impact of low hemoglobin levels and anemia on disability, physical performance decline, decreased muscle strength, and diminished HRQL has also been reported [45]. In a representative population of older individuals in North Carolina, anemia was associated with lower physical function (evaluating basic personal maintenance tasks, such as toileting and bathing, and the ability to function in society, such as shopping and handling money) and cognitive function (using the Short Portable Mental Status Questionnaire) and predicted further functional decline (both cognitive and physical) over a 4-year period [46]. In a recent study on older Iranians from the Bushehr Elderly Health program [47], a significant relationship was found between anemia and physical function variables such as mean handgrip, relative handgrip, and walking speed. An exploratory double-blind randomized intervention trial in older people with anemia found an improvement in hemoglobin levels with a treatment that was associated with corresponding improvement in fatigue and other HRQL outcomes [48]. Another recent study showed that anemia is associated with frailty in older women, lending further support to the potential role of anemia in the evolution to functional decline [49]. Finally, treatment of iron deficiency with intravenous iron infusions for patients with heart failure has demonstrated benefits in quality of life and hospitalizations [29].

Anemia has a negative impact on the circulatory system and leads to the formation of atherosclerotic changes in the vessels [50]. Adequate cerebral oxygenation depends on both arterial oxygen content, determined by the hemoglobin, and blood viscosity, conditioned by the hematocrit [51]. Reduced oxygenation can lead to hypoxia and subsequent inflammation with deleterious effects on neurons [52]. One of the most affected regions could be that implicated in executive function, since it is a domain usually involved in vascular cognitive impairment [53]. Brain imaging findings suggest that white matter structural connectivity, cerebral perfusion, and, potentially, microbleeds may act as pathophysiologic substrates in the hemoglobin–cognition function relationship [54]. Further investigation is needed to better comprehend the physiological role of hemoglobin in brain health.

Patients with both diabetes and anemia have more comorbidities than nonanemic diabetic patients, are more often hospitalized, require medical procedures more frequently, and are at a higher risk of death. Likewise, the prevalence of anemia in subjects with diabetes may be two- to three-times higher than in subjects without [10]. However, the coexistence of anemia and diabetes is not often discussed in the literature, and therefore this problem is not sufficiently recognized. Our study extends previous findings demonstrating an interaction between the coexistence of anemia and diabetes that leads to an exacerbated negative impact on certain executive functions. In our study population, more than half of the participants with anemia were also subjects with diabetes (55%), while only one-quarter of those without anemia were subjects with diabetes (31%). In turn, 7% of subjects with diabetes and 3% of those without diabetes, respectively, had anemia.

Mechanisms potentially leading to anemia development in subjects with diabetes are as follows: erythropoietin deficiency and/or resistance, iron deficiencies (resulting from reduced dietary intake, impaired enteral absorption, blood loss), and proteinuria (with loss of transferrin or erythropoietin) [55]. Hyperglycemia and obesity are important contributors to inflammation, resulting in erythropoietin deficiency or a lack of iron availability for erythropoiesis [56]. Erythropoietin, apart from affecting hematopoiesis, has several other metabolic effects, and erythropoietin receptors have been detected in various tissues, including brain tissue [57].

Both type 1 and type 2 diabetes mellitus have been associated with reduced performance in numerous domains of cognitive function. The exact pathophysiology of cognitive dysfunction in diabetes is not completely understood, but it is likely that hyperglycemia, vascular disease, hypoglycemia, and insulin resistance play significant roles [58]. The co-occurrence of both diabetes and anemia, each independently impacting neuropsychological functions and quality of life in distinct ways, may help elucidate the amplified negative effects observed when both conditions are present. However, further studies to understand the key psychopathological pathways of the coexistence of anemia and diabetes and their impact on cognition and HRQL are necessary.

Our study has limitations. The relatively modest sample size, particularly the limited number of anemic subjects, impacts the statistical power of the analyses. Due to the cross-sectional design of the analysis, reverse causality cannot be ruled out, which prevents causal inferences from being made. The possibility that anemia might be just a marker of diseases and processes associated with chronic cerebral hypoperfusion in the prefrontal cortex or neurodegenerative processes such as inflammation cannot be excluded. Therefore, whether red blood cell levels are directly responsible for the impaired cognition and physical HRQL or whether the associations can be explained by underlying or concomitant vascular or metabolic changes warrant further investigations to fill the methodological gaps of this study due to its cross-sectional nature.

We were unable to provide a more in-depth exploration of the connection between particular types of anemia, cognitive function, and diabetes due to the absence of data regarding the root causes of anemia. While red blood cells are indicators of the blood’s oxygen-carrying capacity, ferritin (a protein that stores iron in the body), serum iron, total Iron-Binding Capacity (TIBC), and transferrin saturation provides information about the body’s iron stores and availability and is particularly useful for identifying iron-deficiency anemia. We did not have measurements of these markers of iron deficiency, which could have reinforced these findings and provided a more comprehensive understanding of the link between anemia and cognition. Nevertheless, the absence of a correlation between dietary iron intake and red blood cell levels in this study may indicate that the underlying cause of anemia could be other than iron deficiency, such as prevalent chronic diseases, bone marrow disorders, or certain medications. Likewise, folate, also known as vitamin B9, plays a role in the production of red blood cells, whose deficiency can be linked to a type of anemia known as folate-deficiency anemia. We did not find, however, significant differences (*p* value 0.71) of folate intake between participants with anemia (353 mcg/d) and without (351 mcg/d). Future research should also include these additional parameters.

The observed association in this study between anemia markers, anemia, cognitive impairment, and quality of life in older participants with overweight/obesity and metabolic syndrome may not necessarily apply to the broader older population. It is important to recognize that study participants likely exhibit elevated levels of cardiometabolic risk markers. Hence, caution should be exercised when attempting to generalize these findings. Nevertheless, this demographic segment represents the population group at the highest risk of anemia and its related complications [29]. Although the analyses were adjusted for chronic disease and other characteristics known to influence both red blood cells and cognition, residual confounding by subclinical conditions or other diseases not measured cannot be ruled out. Likewise, potential confounding mediators or modulator variables, such as inflammatory factors (e.g., C-reactive-protein) or markers of renal function (e.g., creatinine), were not accounted for in this study due to the unavailability of these data. Incorporating data on these additional metabolic indicators and broadening the sample to include individuals without obesity or metabolic syndrome would enhance our comprehensive understanding of the impact of anemia on cognitive function. 

Lastly, while we employed a wide array of the most common neuropsychological tests used in research—with each targeting distinct domains of cognitive and executive functions—certain cognitive domains, particularly those involving high-level cognitive and social skills, may be not adequately represented. Likewise, some test procedures often diverge from real-world activities (ecological validity), and our understanding of the intricate neural networks activated during cognitive tasks is still in its infancy. Also, paper and pencil neuropsychological tests are more likely to suffer from some level of subjectivity and variability compared with computerized neuropsychological tests. 

## 5. Conclusions

In this cross-sectional study of older adults at high risk of cardiovascular disease, lower anemia markers and anemia were associated with poorer cognitive function and worse scores in different domains of executive function and a poorer physical component of quality of life (physical function, role limitations due to physical health problems, bodily pain, vitality, and general health). However, red cell levels were not associated with quality of life at the mental level. The coexistence of diabetes in anemic patients increases the impact of anemia on cognitive function and quality of life (statistically significant interaction).

Further research is required to gain a comprehensive understanding of the mechanisms underlying the apparent associations between anemia or low levels of iron biomarkers and compromised cognition and quality of life. Interestingly, dietary iron intake appears to have no influence on these outcomes. To establish definitive guidelines for clinical practice, it is imperative that this hypothesis and our findings undergo comprehensive testing through methodologically rigorous studies designed to mitigate the potential for reverse causation. These findings warrant further evaluation of a wider use of iron metabolism biomarkers for clinical decision making in patients.

## Figures and Tables

**Table 1:** Descriptive variables according to anemia status. Cross-sectional assessment in the PREDIMED-PLUS study including 6117 participants.

	Non-Anemic *n* = 5865	Anemic ^1^ *n* = 252	*p*-Value
Hemoglobin (g/dl)	14.7 (1.7)	11.3 (1.7)	<0.001
Age (years)	65.5 (4.9)	66.9 (4.8)	<0.001
Female sex (%)	47.7	56.3	0.01
Maximum attained educational level (%)			<0.001
Primary school (%)	49.0	61.9	
Secondary school (%)	28.8	22.2	
College (%)	22.3	15.9	
BMI (kg/m^2^)	32.5 (3.4)	33.0 (3.6)	0.01
Glucose (mg/dL)	113 (29)	121 (37)	<0.001
TyG index ^2^	8.93 (0.51)	8.98 (0.56)	0.92
Total iron intake (mg/d)	16.4 (4.0)	16.3 (4.4)	0.49
Heme iron intake (mg/d)	1.94 (0.69)	1.90 (0.72)	0.35
Vitamin B12 intake (mcg/d)	9.80 (4.4)	10.5 (5.9)	0.02
Folic acid intake (mcg/d)	351 (102)	353 (106)	0.71
Diabetes (%)	31.4	55.2	<0.001
Hypertension (%)	94.3	95.2	0.53
Hypercolesterolemia (%)	78.9	80.2	0.63
Depression (%)	20.5	23.8	0.20
Cancer (%)	6.87	9.92	0.06
Adherence to Mediterranean diet (17-point score)	8.58 (2.66)	8.71 (2.56)	0.45
Alcohol consumption (g/d)	11.3 (15.3)	8.8 (12.9)	0.01
Smoking			0.01
Current	13.2	7.94	
Former	43.6	40.9	
Never	43.3	51.2	
Physical activity (METs min/wk)	2467 (2309)	2220 (2162)	0.10
Sleep (hours/d)	7.09 (1.21)	7.01 (1.3)	0.33

Continuous variables presented as mean ± standard deviation and categorical variables as percentage and number of participants. *p*-values were obtained using chi square test for categorical variables and T Student for continuous variables. ^1^ Anemia was defined according to the World Health Organization (WHO) criteria of hemoglobin concentrations below 13 g/dL for men and below 12 g/dL for women. ^2^ TyG index = triglyceride and glucose index. The TyG index is calculated as: ln [fasting triglycerides (mg/dL) × fasting plasma glucose (mg/dL)]/2.

**Table 2:** Cross-sectional associations of anemia status with cognitive and executive functions and health-related quality of life.

	Mean (SD) by Anemia Status					Adjusted-Mean Difference (β) (95% CI)	
	Non-Anemic		Anemic		*p*-Value	Anemic vs. Non-Anemic	
Cognitive function	Mean (SD)	*n*	Mean (SD)	*n*		Model 1	Model 2
MMSE ^1^ (Total score)	28.2 (1.90)	5952	27.7 (2.46)	245	<0.001	−0.40 (−0.64, −0.17) *	−0.30 (−0.53, −0.06) *
Executive function	Mean (SD)	*n*	Mean (SD)	*n*		Model 1	Model 2
Trail Making Test: A, total time (s) ^2^	53.0 (28.6)	6066	59.7 (34.2)	246	<0.001	3.95 (0.44, 7.46) *	2.11 (−1.31, 5.53)
Trail Making Test: B, total time (s) ^2^	130 (72.6)	6047	156 (83.6)	245	<0.001	18.5 (9.64, 27.37) *	12.1 (3.69, 20.5) *
Semantic verbal fluency of animals: total	16.1 (4.93)	6077	15.2 (4.53)	247	0.003	−0.53 (−1.13, 0.06)	−0.21 (−0.78, 0.36)
Phonological verbal fluency of letter P: total	12.2 (4.54)	6077	11.2 (4.34)	247	<0.001	−0.77 (−1.33, −0.20) *	−0.38 (−0.90, 0.15)
Digit total score (forward + backward)	13.8 (4.12)	4181	12.7 (3.86)	168	<0.001	−0.66 (−1.27, −0.06) *	−0.44 (−1.02, 0.13)
Clock Drawing Test	5.93 (1.23)	5706	5.77 (1.36)	246	0.029	−0.10 (−0.26, 0.05)	−0.06 (−0.21, 0.10)
SF36-HRQL test ^3^	Mean (SD)	*n*	Mean (SD)	*n*		Model 1	Model 2
Aggregated physical dimension	45.4 (8.82)	5857	41.7 (9.64)	239	<0.001	−3.15 (−4.26, −2.05) *	−2.53 (−3.61, −1.45) *
Aggregated mental dimension	51.1 (10.5)	5857	50.2 (11.3)	239	0.178	−0.81 (−2.14, 0.53)	−0.18 (−1.46, 1.10)

Anemia was defined according to the World Health Organization (WHO) criteria of hemoglobin concentrations below 13 g/dL for men and below 12 g/dL for women. The *n* available for each test may differ due to missing data in some tests. Values marked with an asterisk (*) indicate that they are statistically significant. ^1^ MMSE: Mini-Mental State Examination. ^2^ For these tests, unlike the others, the lower the score (time), the better the cognitive function. ^3^ Short Form-36 Health Survey for evaluating health-related quality of life. Model 1 adjusted for sex, age, center, and vitamin B12 and folate. Model 2 further adjusted for BMI, educational level, tobacco use, physical activity, adherence to a Mediterranean diet (score of 17 points), hours of sleep, alcohol consumption, hypertension, hypercholesterolemia, diabetes, cancer, and depression.

**Table 3:** Cross-sectional associations of anemia status with cognitive and executive functions, and quality of life by prevalent diabetes.

	Adjusted-Mean Difference (β) (95% CI) Anemic vs. Non-Anemic		
	Subjects with Diabetes *n* = 1983	Subjects without Diabetes *n* = 4134	
Anemia	139 (7%)	113 (3%)	
Cognitive function			P-interaction
MMSE ^1^ (Total score)	−0.26 (−0.60, 0.08)	−0.34 (−0.67, −0.00) *	0.793
Executive function			P-interaction
Trail Making Test: A, total time (seconds) ^2^	5.87 (0.85, 10.9) *	−2.60 (−7.46, 2.25)	0.009
Trail Making Test: B, total time (seconds) ^2^	21.4 (9.34, 33.4) *	0.54 (−11.6, 12.7)	0.007
Semantic verbal fluency of animals: total	−0.21 (−0.97, 0. 56)	−0.17 (−1.02, 0.69)	0.912
Phonological verbal fluency of letter P: total	−0.83 (−1.52, −0.13) *	0.17 (−0.62, 0.95)	0.057
Digit total score (forward + backward)	−0.41 (−1.21, 0.39)	−0.49 (−1.32, 0.35)	0.956
Clock Drawing Test	−0.10 (−0.31, 0.12)	−0.02 (−0.25, 0.21)	0.697
SF36-HRQL test ^3^			
Aggregated physical dimension	−2.82 (−4.28, −1.36) *	−2.13 (−3.72, −0.55) *	0.517
Aggregated mental dimension	−0.85 (−2.68, 0.97)	0.67 (−1.16, 2.51)	0.131

The *n* available for each test may differ due to missing data in some tests. Values marked with an asterisk (*) indicate that they are statistically significant. ^1^ MMSE: Mini-Mental State Examination. ^2^ For these tests, unlike the others, the lower the score (time), the better the cognitive function. ^3^ Short Form-36 Health Survey for evaluating health-related quality of life. Estimations adjusted for sex, age, center, vitamin B12 and folate, BMI, education level, tobacco use, physical activity, adherence to a Mediterranean diet (score of 17 points), hours of sleep, alcohol consumption, hypertension, hypercholesterolemia, diabetes, cancer, and depression.

**Table 4:** Cross-sectional associations of total iron intake (mg/day) with cognitive and executive functions and quality of life.

	Adjusted-Mean Difference (β) (95% CI)		
	Total Energy-Adjusted Iron Intake		
	Tertile 1	Tertile 2	Tertile 3
Total iron intake (mg/day), mean (SD)	13.9 (3.0)	15.9 (2.9)	19.6 (3.7)
Cognitive function			
MMSE ^1^ (Total score)	0 (ref.)	0.04 (−0.07, 0.16)	−0.10 (−0.24, 0.03)
Executive function			
Trail Making Test: A, total time (seconds) ^2^	0 (ref.)	0.29 (−1.34, 1.91)	2.83 (0.87, 4.78) *
Trail Making Test: B, total time (seconds) ^2^	0 (ref.)	0.21 (−3.81, 4.24)	5.90 (1.06, 10.7) *
Semantic verbal fluency of animals: total	0 (ref.)	−0.09 (−0.37, 0.18)	0.02 (−0.31, 0.35)
Phonological verbal fluency of letter P: total	0 (ref.)	−0.18 (−0.43, 0.07)	0.05 (−0.25, 0.35)
Digit total score (forward + backward)	0 (ref.)	−0.09 (−0.38, 0.19)	0.02 (−0.33, 0.36)
Clock Drawing Test	0 (ref.)	0.02 (−0.05, 0.10)	−0.00 (−0.09, 0.09)
SF36-HRQL test ^3^			
Aggregated physical dimension	0 (ref.)	−0.12 (−0.64, 0.41)	0.01 (−0.61, 0.64)
Aggregated mental dimension	0 (ref.)	0.35 (−0.27, 0.97)	0.04 (−0.70, 0.78)

Values marked with an asterisk (*) indicate that they are statistically significant. ^1^ MMSE: Mini-Mental State Examination. ^2^ For these tests, unlike the others, the lower the score (time), the better the cognitive function. ^3^ Short Form-36 Health Survey for evaluating health-related quality of life. Estimations adjusted for sex, age, center, vitamin B12 and folate, BMI, educational level, tobacco use, physical activity, adherence to a Mediterranean diet (score of 17 points), hours of sleep, alcohol consumption, hypertension, hypercholesterolemia, diabetes, cancer, and depression.

## Data Availability

The use of the dataset supporting the conclusions of this article is restricted due to the signed consent agreements around data sharing, which only allow access to external researchers for studies following the project purposes. Requestors wishing to access the data used in this study can make a request to the Steering Committee (predimed_plus_scommittee@googlegroups.com). The request will then be passed to the Steering Committee for deliberation.

## Supplementary Materials

The following supporting information can be downloaded at: https://www.mdpi.com/article/10.3390/nu15204440/s1, File S1: Description of neurogical tests; File S2—Table S1: Cross-sectional associations of anemia markers with cognitive function, executive function, and health-related quality of life; File S2—Table S2: Cross-sectional associations of anemia status and anemia markers with each physical dimension of the quality-of-life test [59,60,61,62,63,64,65].
