# Supplementary material for: Dietary Iron, Anemia Markers, Cognition, and Quality of Life in Older Community-Dwelling Subjects at High Cardiovascular Risk
Source: Nutrients. 2023 Oct 19;15(20):4440. doi: 10.3390/nu15204440 (PMC10610130; doi:10.3390/nu15204440)
Supplement: Supplementary file 1 [file nutrients-15-04440-s001.zip › nutrients-2567937-supplementary/Supplementary Materials File S1.pdf]

Supplemental Material S1

The **Mini-Mental State Examination (MMSE**) validated for the Spanish population was used as a general cognitive screening test, which is an assessment of general cognitive function. The MMSE is divided into two sections, the first of which requires vocal responses only and covers orientation, memory, and attention, being the maximum score 21. The second part tests the ability to name, follow verbal and written commands, write a sentence spontaneously, and copy a complex polygon similar to a Bender-Gestalt figure, with a maximum possible score of 9. A high MMSE score indicates absence of cognitive decline [1].

The **Clock Drawing Test (CDT**) was used as a cognitive screening tool and the 7-point version, currently used for elderly Spanish populations, was performed [2]. Higher scores indicate better performance. The CDT assesses visuospatial, visuocontruction and memory capacities, as well as verbal and numerical knowledge [3].

The **Digit Span Test (DST)** of the WAIS-III Spanish version measuring attention and memory functions was used [4]. The Digit Span Test forward version (DST-f) requires participants to verbally repeat in the same order as provided, a number sequence, varying from 2 to 9 numbers, being representative of immediate memory. The DST backward version (DST-b) requires participants to verbally repeat in reverse order a number sequence, varying from 2 to 8 numbers, being representative of working memory function [5]. For both tests, the task is finished when the participant fails two attempts consecutively.

The **Spanish Verbal Fluency Tests** (VFTs) were used to assess language and executive function [6]. Specifically, we used the semantic VTF animal category version (VFT-a) and the phonemic VFT letter “p” version (VFT-p). Participants were instructed to say the maximum number of words related to the semantic field of animals for VFT-a and the maximum number of words starting with the letter “p” for VFT-p, having a time limit for

both tests of 60 seconds. The total score was obtained from the row of properly stated words. The semantic VFT presents more influence on verbal abilities, whereas the phonologic VFT presents more influence on executive control ability [7].

The **Trail Making Test (TMT)** with normative data provided for the Spanish population was used [8]. The TMT consists of 25 circles spread over a sheet of paper and contains part A (TMT-A) and part B (TMT-B). In TMT-A, participants were asked to connect consecutive numbers (1–2–3–…) in the correct order by drawing a line. In TMT-B, they were asked to connect consecutive numbers and letters in an alternating numeric and alphabetic sequence (1–A–2–B–3–C–…). Each part is scored according to the time taken to complete the task, where more time spent indicates poorer performance. TMTs discriminate cognitive dysfunction [9] and TMT-A assesses attention and processing speed capacities [8] whereas TMT-B requires more executive function abilities such as cognitive flexibility [10].

REFERENCES

1. Folstein MF, Folstein SE, McHugh PR. “Mini-mental state”. A practical method for grading the cognitive state of patients for the clinician. J Psychiatr Res. 1975;12(3):189–98.

2. del Ser Quijano T, García de Yébenes MJ, Sánchez Sánchez F, Frades Payo B, Rodríguez Laso Á, Bartolomé Martínez MP, et al. Evaluación cognitiva del anciano. Datos normativos de una muestra poblacional española de más de 70 años. Med Clin (Barc). 2004;122(19):727–40.

3. Shulman KI. Clock-drawing: is it the ideal cognitive screening test? Int J Geriatr Psychiatry. 2000;15(6):548–61.

4. TEA SA. WAIS-III: Escala de inteligencia de Wechsler para Adultos. [WAIS-III: Wechsler adult Intelligence scale. Third version]. TEA. Madrid; 1999.

5. Wechsler D. Wechsler Memory Scale - Revised. Manual. The Psychological Corporation. San Antonio, TX; 1987.

6. Peña-Casanova J, Quiñones-Úbeda S, Gramunt-Fombuena N, Quintana-Aparicio M, Aguilar M, Badenes D, et al. Spanish multicenter normative studies (NEURONORMA project): Norms for verbal fluency tests. Arch Clin Neuropsychol. 2009;24(4):395–411.

7. Shao Z, Janse E, Visser K, Meyer AS. What do verbal fluency tasks measure? Predictors of verbal fluency performance in older adults. Front Psychol. 2014;5(772).

8. Llinàs-Reglà J, Vilalta-Franch J, López-Pousa S, Calvó-Perxas L, Torrents Rodas D, Garre-Olmo J. The Trail Making Test. Assessment. 2017;24(2):183–96.

9. Reitan RM. The relation of the Trail Making Test to organic brain damage. J Consult Psychol. 1955;19(5):393–4.

10. Kortte KB, Horner MD, Windham WK. The Trail Making Test, Part B: Cognitive Flexibility or Ability to Maintain Set? Appl Neuropsychol. 2002;9(2):106–9.
