# Supplementary material for: Dietary Iron, Anemia Markers, Cognition, and Quality of Life in Older Community-Dwelling Subjects at High Cardiovascular Risk
Source: Nutrients. 2023 Oct 19;15(20):4440. doi: 10.3390/nu15204440 (PMC10610130; doi:10.3390/nu15204440)
Supplement: Supplementary file 1 [file nutrients-15-04440-s001.zip › nutrients-2567937-supplementary/Supplementary Materials File S2.pdf]

Supplemental Material S2

Supplemental Table S1. Cross-sectional associations of anemia markers with cognitive function, executive function, and health-related quality of life.

	Adjusted-mean difference (β) (95% CI)					
	**Hemoglobin** (g/dl)*		**Hematocrit** (%)*		**MCV**[4] (fL)*	
**Cognitive function**	**Model 1**	**Model 2**	**Model 1**	**Model 2**	**Model 1**	**Model 2**
Minimental test[1] (Total score)	0.08 (0.03, 0.13)	0.06 (0.01, 0.11)	0.09 (0.04, 0.14)	0.06 (0.01, 0.11)	0.06 (0.02, 0.11)	0.04 (-0.00, 0.09)
**Executive function**	**Model 1**	**Model 2**	**Model 1**	**Model 2**	**Model 1**	**Model 2**
Trail Making Test: A, total time (seconds)[2]	0.51 (-0.24, 1.26)	0.85 (0.12, 1.58)	0.01 (-0.75, 0.77)	0.41 (-0.34, 1.16)	-0.04 (-0.74, 0.66)	0.32 (-0.36, 1.00)
Trail Making Test: B, total time (seconds)[2]	-3.33 (-4.13, -0.54)	-1.75 (-3.57, 0.07)	-3.43 (-5.35, -1.51)	-2.01 (-3.85, -0.17)	0.45 (-2.21, 1.32)	0.67 (-0.01, 2.34)
Semantic verbal fluency of animals: total	0.19 (0.07, 0.32)	0.02 (-0.10, 0.14)	0.19 (0.07, 0.32)	0.12 (-0.01, 0.24)	0.13 (0.01, 0.25)	0.06 (-0.06, 0.17)
Phonological verbal fluency of letter P: total	0.12 (0.00, 0.24)	0.05 (-0.06, 0.16)	0.19 (0.07, 0.31)	0.09 (-0.03, 0.20)	0.16 (0.05, 0.27)	0.08 (-0.03, 0.18)
Digit total score (forward + backward)	0.05 (-0.10, 0.19)	-0.02 (-0.16, 0.12)	0.05 (-0.10. 0.19)	0.03 (-0.09, 0.16)	0.08 (-0.05, 0.21)	-0.05 (-0.16, 0.06)
Clock Drawing Test	0.03 (-0.01, 0.06)	0.02 (-0.02, 0.05)	0.02 (-0.01, 0.06)	0.01 (-0.02, 0.05)	0.01 (-0.02, 0.04)	0.00 (-0.03, 0.03)
**SF36-HRQL test**[3]	**Model 1**	**Model 2**	**Model 1**	**Model 2**	**Model 1**	**Model 2**
Aggregated physical dimension	0.66 (0.41, 0.91)	0.50 (0.25, 0.74)	0.41 (0.17, 0.66)	0.28 (0.04, 0.52)	0.36 (0.14, 0.58)	0.31 (0.10, 0.52)
Aggregated mental dimension	0.15 (-0.15, 0.45)	-0.02 (-0.31, 0.27)	0.27 (-0.02, 0.57)	0.17 (-0.11, 0.46)	0.12 (-0.14, 0.38)	0.10 (-0.15, 0.35)

* Per 1-standard deviation increment

[1]MMSE: Mini-Mental State Examination

[2] For these tests, unlike the others, the lower the score (time), the better the cognitive function.

[3] Short Form-36 Health Survey for evaluating the health-related quality of life.

[4] Medium corpuscular volume

Model 1 adjusted for sex, age, center, vitamin B12 and folic acid.

Model 2 further adjusted for BMI, educational level, tobacco use, physical activity, adherence to a Mediterranean diet (score of 17 points), hours of sleep, alcohol consumption, hypertension, hypercholesterolemia, diabetes cancer and depression

Supplemental Table S2. Cross-sectional associations of anemia status and red blood cells with each physical dimension of the quality of life test.

	Adjusted-mean difference (β) (95% CI)			
	**Anemia**	**Hemoglobin***	**Hematocrit***	**MCV[2]***
**Physical dimension of the SF36-HRQL test**				
Physical function	-4.60 (-6.79, -2.40)	0.68 (0.21, 1.15)	0.81 (0.33, 1.30)	0.33 (-0.11, 0.77)
Role physical[1]	-7.74 (-12.7, -3.42)	1.45 (0.50, 2.41)	1.33 (0.08, 1.47)	1.23 (0.37, 2.09)
Bodily pain	-3.38 (-6.54, -0.23)	1.19 (0.51, 1.86)	0.78 (0.39, 1.82)	0.54 (-0.09, 1.17)
Vitality	-2.65 (-5.16, -0.13)	0.77 (0.22, 1.32)	0.49 (-0.07, 1.05)	0.35 (-0.15, 0.85)
General health	-4.91 (-7.17, -2.66)	0.78 (0.28, 1.28)	0.06 (-0.44, 0.56)	0.74 (0.29, 1.19)

Anemia was defined according to the World Health Organization (WHO) criteria of hemoglobin concentrations below 13 g/dL for men and below 12 g/dL for women.

* Per 1-standard deviation increment

[1]Role limitations due to physical health problems

[2] Medium corpuscular volume

Estimations adjusted for sex, age, center, vitamin B12 and folic acid, BMI, educational level, tobacco use, physical activity, adherence to a Mediterranean diet (score of 17 points), hours of sleep, alcohol consumption, hypertension, hypercholesterolemia, diabetes cancer and depression.
